# Environmental determinants of total IgE among school children living in the rural Tropics: importance of geohelminth infections and effect of anthelmintic treatment

**DOI:** 10.1186/1471-2172-9-33

**Published:** 2008-06-27

**Authors:** Philip J Cooper, Neal Alexander, Ana-Lucia Moncayo, Susana M Benitez, Martha E Chico, Maritza G Vaca, George E Griffin

**Affiliations:** 1 Laboratorio de Investigaciones FEPIS, Quininde, Esmeraldas Province, Ecuador; 2 Instituto de Microbiologia, Universidad San Francisco de Quito, Quito, Ecuador; 3 Centre for Infection, St George's University of London, London, UK; 4 Infectious Disease Epidemiology Unit, London School of Hygiene and Tropical Medicine, London, UK

## Abstract

**Background:**

The environmental factors that determine the elevated levels of polyclonal IgE observed in populations living in the Tropics are poorly understood but may include geohelminth infections. We investigated the association between geohelminth infections and total IgE levels in school children in rural tropical Ecuador, and assessed the effect on IgE of repeated anthelmintic treatments over a period of 12 months. The study was nested within a cluster-randomized study that randomized 68 schools to receive either 400 mg of albendazole every 2 months over a year or no treatment. We studied random samples of children completing follow-up and representing four groups stratified by the presence of geohelminth infection at baseline and treatment allocation. We measured levels of total IgE and anti-*A. lumbricoides *IgG (used as a measure of past and current geohelminth infectious exposure) in blood samples collected at the start of the study and after 12 months.

**Results:**

We observed elevated levels of total IgE (compared to standard reference values) at the start of the study in this population of school children (geometric mean, 1,004 IU/mL, range 12 to 22,608 IU/mL)) and baseline IgE levels were strongly associated with parameters of geohelminth infection but not with age, nutritional and socioeconomic status. After 12 months, levels of IgE fell significantly in the treatment (by 35.1%) and no treatment (by 10.4%) groups, respectively, but the fall was significantly greater in the treatment group. Falls in IgE were independently associated with albendazole treatment, having a baseline geohelminth infection and with high baseline levels of anti-*A. lumbricoides *IgG. Increases in IgE at 12 months were associated with the presence of geohelminth infections and increasing levels of anti-*A. lumbricoides *IgG at 12 months independent of treatment allocation.

**Conclusion:**

The data provide evidence that geohelminth infections are an important determinant of total IgE in school children in the rural Tropics and that periodic anthelmintic treatments over 12 months are associated with reductions in IgE. The failure of anthelmintic treatment to reduce IgE levels to that considered normal in industrialized countries may be attributed to continued exposure of children to geohelminths or to the effects of infections in early life in programming a long-lasting Th2-biassed immunity.

## Background

Levels of polyclonal IgE in the peripheral blood of poor rural [1] and urban populations [2-4] living in the tropics are highly elevated compared to levels observed in wealthier urban populations in the same countries [5] or in populations living in industrialized countries [6]. Total IgE levels in industrialized countries are affected by age [7] and genetic factors [8] and are associated with ethnicity and socioeconomic status [9]. The causes of the high circulating levels of IgE in poor tropical populations are poorly understood but have been attributed again to genetic factors [10,11] and also to infections with geohelminth (intestinal helminth) parasites [12].

Geohelminth parasites are the most prevalent of all helminth infections and are estimated to infect approximately 2 billion humans worldwide [13]. Important geohelminth parasites include *Ascaris lumbricoides*, *Trichiuris trichiura*, hookworm, and *Strongyloides stercoralis*. Infections with these parasites induce strong immunological responses in infected humans that are associated with the production of type 2 cytokines including IL-4, IL-5, and IL-13 [14,15]. The high levels of polyclonal IgE observed in populations living in areas that are endemic for geohelminth parasites may be caused by non-specific stimulation of polyclonal IgE by geohelminths through an IL-4-dependent mechanism [16]. A possible causal association between geohelminth infections and levels of total IgE may be suggested by the observation that IgE levels decline after effective anthelmintic treatment [16-18].

The objective of the present study was to investigate the association between total IgE and geohelminth infections and other risk factors in school children living in an area of the rural tropics that is highly endemic for geohelminth parasites, and to examine the effect of repeated anthelmintic treatments for a period of a year on levels of total IgE.

## Methods

### Subjects and sampling

School children attending schools in rural areas of Pichincha Province in Ecuador were sampled within a cluster-randomized study that examined the effect of albendazole treatment on the prevalence of allergy. The study design is described in detail elsewhere [19]. Briefly, mestizo children attending the second to seventh year of primary education in 68 rural schools were recruited. Schools were randomized to receive either albendazole (single doses of 400 mg provided every 2 months for a period of 12 months [total of 7 treatments] or no treatment. Albendazole treatments were directly observed and a placebo was not used in the no-treatment group. A total of 1,632 children (68.8% of 2,373 children that were recruited at the start of the study – follow-up rates were similar in treatment (67.4%) and no-treatment (70.1%) groups) – completed follow-up at 12 months in the 68 schools but 23 of these children with no baseline stool results were excluded from the analysis. Four samples of each of 100 individuals were selected randomly from the remaining 1,609 children to represent the four possible combinations of treatment and baseline infection as follows: 1) infection/no treatment (sampling fraction, 100/622) – children in the no treatment group that were infected at the beginning of the study with geohelminths; 2) no infection/no treatment (100/214) – children in the no treatment group that were not infected with geohelminths at the beginning of the study; 3) infection/treatment (100/543) – children in the treatment group that were infected with geohelminths at the beginning of the study; and 4) no infection/treatment (100/230) – children in the treatment group that were not infected with geohelminths at the beginning of the study. The study protocol was approved by the Ethics Committees of St George' University of London, London, UK, and the Hospital Pedro Vicente Maldonado, Pichincha Province, Ecuador. Informed written consent to participate in the study was obtained from a parent or guardian of all children.

### Sample collection and analysis

Stool samples were collected from children at the beginning of the study (i.e. before receiving the 1^st ^dose of albendazole) and at 12 months (i.e. before receiving the 7^th ^dose of albendazole). Blood samples (3 mL) were drawn from the antecubital fossa into Vacutainers (Becton Dickinson) containing sodium heparin as anticoagulant at the beginning of the study and 7 days after receiving the 7^th ^dose of albendazole. The blood was centrifuged and the plasma aliquotted and stored at -20C until use. Total IgE was measured in IU/mL as described previously [1]. Levels of *A. lumbricoides *– specific total IgG antibodies were measured by an antibody-capture ELISA as described [14]. Briefly, plates were coated with a PBS-soluble extract of *A. lumbricoides *adult female worms (obtained by expulsion after anthelmintic treatment) at 2.5 μg/mL in carbonate buffer. After incubation with plasma samples diluted at 1/100, the plates were incubated with alkaline phosphatase conjugated anti-human IgG (Jackson ImmunoResearch), developed with p-nitrophenyl phosphate (Sigma) in sodium carbonate buffer, and read on a microtiter plate reader. Unknown values were interpolated from a standard curve derived from pools of positive serum samples, and antibody levels were expressed as arbitrary units. The cut-off for a positive serologic test for *Ascaris lumbricoides*-specific IgG was defined as mean plus 3 standard deviations of antibody levels obtained from 8 individuals resident in the town of Quinindé in Esmeraldas Province with no previous history of geohelminth infection. Stool samples were examined using the modified Kato-Katz and formol-ether acetate concentration methods as described [20].

### Statistical analysis

The random samples that comprised the four study groups were generated using computer software (Stata 7, Stata Corporation, College Station, Texas, USA). Children were analysed according to their original treatment allocation. The primary outcomes were baseline levels of IgE and fold change in total IgE expressed as a percentage (100*posttreatment IgE/pretreatment IgE). Secondary outcomes were baseline levels of anti-*A. lumbricoides *IgG and changes in anti-*A. lumbricoides *IgG expressed as a percentage also. All analyses used logarithmically transformed data for the dependent continuous variables of baseline IgE and changes in IgE. The results of transformed data for IgE parameters were back-transformed to compare explanatory variables in terms of ratios (fold differences) of geometric means. Changes in IgE levels compared to baseline were assessed using the one-sample t test. Inter-group differences for IgE outcomes were assessed using one-way analysis of variance (for 4 group comparisons) and Student's t test (for 2 group comparisons). Multivariable analyses for IgE and anti-*A. lumbricoides *IgG outcomes used linear regression and controlled for the *a priori *confounding factors, age, sex, body mass index, socioeconomic level and household crowding. Clustering by school was adjusted for using the sandwich estimator ('robust cluster' in STATA). Four models were used to explore the associations between geohelminth infections and the primary IgE outcomes. The models were chosen to investigate the independent effects on these outcomes of different parameters of geohelminth infections including the presence of any infection and exposure to any infection, the presence of infections with different geohelminth parasites, and infection intensities with *A. lumbricoides *and *T. trichiura *(the predominant geohelminth parasites in the study area). The models were: Model 1 – association with any geohelminth infection; Model 2 – associations with individual geohelminth parasite infections; Model 3 – associations with infection intensities with *A. lumbricoides *and *T. trichiura*; and Model 4 – associations with geohelminth prevalence, and exposure to geohelminth parasites. Exposure to geohelminth infection was measured by anti-*A. lumbricoides *IgG antibodies – because there is extensive immunologic cross-reactivity between antigen preparations from different geohelminth parasites [21], the presence of IgG antibodies to *A. lumbricoides *is likely to indicate past and or present exposures to geohelminth infections including *A. lumbricoides *and antibody levels are likely to be associated with the intensities of current and recent infections. Linear regression models for changes in IgE included also parameters for geohelminth infection at 12 months. The secondary outcomes, baseline levels and changes in anti-*A. lumbricoides *IgG, were logarithmically transformed and analysed using Model 3 only. Bonferroni corrections were used to conservatively reduce the probability of Type I errors in those multivariable analyses involving multiple geohelminth-associated variables – the original significance level of 5% was divided by the number of such variables in each analysis to obtain an adjusted significance level for them. Therefore, the multiples used for Models 1, 2, 3, and 4 for total IgE and changes in total IgE were: 1, 4, 6, and 3 and 2, 8, 12, and 6, respectively, Bonferroni corrections for multivariable analyses of baseline and changes in anti-*A. lumbricoides *IgG were as for Model 3. Socioeconomic status was calculated as a score divided into quartiles and based on paternal and maternal educational level (categorized as illiterate [lowest] to completed higher education [highest]) and occupation (i.e. categorized as day-labourer [lowest] to professional [highest]), and the number of material goods in the household (radio, television, refrigerator, and sound system). Each variable was assigned a score and the range of scores was divided into quartiles numbered 1 to 4 representing the richest to poorest quartiles, respectively. Each individual was assigned a final quartile socioeconomic level score of 1 to 4. Geohelminth infection intensities were stratified into three groups (negative, and two positive groups [above and below the median]), and anti-*A. lumbricoides *IgG, when used as an independent variable, into tertiles. Tests for trend were included in the regression models by allocating ordinal scores (0, 1 and 2) to the categories in ascending order, and retaining the other model covariates. Analyses were done with Stata version 8.0.

## Results

### General characteristics of study groups

The baseline characteristics of the study groups are shown in Table 1. The mean age, sex distribution, and mean levels of crowding (number of persons per sleeping room) were similar between the 4 groups. Socioeconomic level was significantly different between the 4 study groups (P < 0.0001) and tended to be lower (i.e. higher score) in infected children in both treatment groups compared to the respective non-infected groups. *A. lumbricoides *and *T. trichiura *infections were the main geohelminth infections present in this population, and the prevalence of all 4 geohelminths present in this sample were similar between the 2 infection groups (infection/no treatment and infection/treatment). A total of 185 of the 200 (93%) individuals in the two treatment groups (infection/treatment and no infection/treatment) received all 7 albendazole treatments, and only 4 individuals received 3 or fewer treatments. Treatment was incomplete in the treatment group as a consequence of failure of a few children to attend the school on treatment days or moving house from a treatment to a no treatment school over the course of the study (3 children). Five individuals in the no treatment groups had documented albendazole treatments during the study as a consequence of moving to a treatment school during the 12-month observation period.

**Table 1:** Baseline characteristics of the four study groups.

Variable	Infection/No treatment (n = 100)	No infection/No treatment (n = 100)	Infection/treatment (n = 100)	No infection/Treatment (n = 100)
Age (years)				
Mean (SD)	9.5 (2.0)	9.3 (1.8)	9.2 (1.6)	9.4 (1.8)
Sex				
Male/Female	55/45	47/43	50/50	54/46
Socioeconomic level				
Mean (SD)	2.6 (1.1)	2.0 (0.9)	2.2 (0.9)	1.9 (0/8)
Body mass index (kg/m2)				
Mean (SD)	15.8 (1.8)	15.9 (2.5)	15.6 (1.5)	16.1 (2.4)
Crowding (persons/room)				
Mean (SD)	2.7 (1.2)	2.4 (1.2)	2.8 (1.1)	2.4 (1.1)
Geohelminth prevalence (%)				
Any	100	0	100	0
*A. lumbricoides*	75	0	73	0
*T. trichiura*	76	0	80	0
Hookworm	19	0	15	0
*S. stercoralis*	3	0	3	0
Intensity, GM (range) epg				
*A. lumbricoides*	5,658 (70–227,500)	0	6,512 (70–182,700)	0
*T. trichiura*	602 (70–40,250)	0	583 (70–13,720)	0
Number of albendazole treatments				
0	98%	97%	0%	0%
1–3	2%	2%	4%	0%
4–6	0%	0%	3%	8%
7	0%	1%	93%	92%

GM – geometric mean. SD – standard deviation. Epg – eggs per gramme of stool. Anthelmintic treatments were with single doses of 400 mg of albendazole.

### Changes in prevalence of geohelminth infections

**Figure 1:**
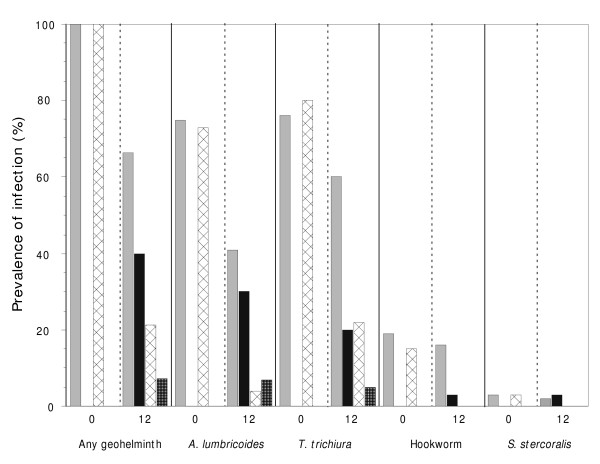
Prevalence of infection with geohelminths during the study. Shown is the prevalence of infections before receiving the 1^st ^dose of albendazole (0 months) and at 12 months (before receiving the 7^th ^dose of albendazole) in the four study groups. Groups are: 1) infection/no treatment (light grey columns), 2) no infection/no treatment (dark grey), 3) infection/treatment (hatched), and 4) no infection/treatment (checked).

Changes in the prevalence of geohelminth infections are shown in Figure 1. The prevalence of infection with any geohelminth parasite declined significantly from 100% to 67% in the infection/no treatment group (P < 0.001) and group from 100% to 24% (P < 0.001) in the infection/treatment group. The treatment effect on geohelminth prevalence at 12 months was statistically significant (OR 0.39, 95% CI 0.29–0.54, P < 0.001), indicating a 61% relative reduction in geohelminth prevalence in the treatment compared to the no treatment group. Anthelmintic treatment was more effective for *A. lumbricoides *than *T. trichiura*. Forty-two percent of individuals in the no infection/no treatment group acquired geohelminth infections over the 12-month period compared to 9% of individuals in the no infection/treatment group. No infections with hookworm and *S. stercoralis *were observed in either of the two treatment groups at 12 months. The decline in geohelminth prevalence in the no treatment group may have been caused by treatment contamination. Anthelmintic treatment is widely available without prescription in pharmacies and dispensaries throughout Ecuador, and mothers frequently administer anti-parasite drugs to their children without medical consultation. Five individuals in the non-treated communities actually moved from treated to non-treated communities over the 12 month study period and had documented treatments with albendazole.

### Factors associated with total IgE at baseline

Geometric mean levels of IgE in the study population were 1,004 IU/mL (range 12–22,608 IU/mL) and were similar between the infection (infection/no treatment, 1,315 IU/mL vs. infection/treatment, 1,566 IU/mL, P = 0.34) and the no infection groups (no infection/no treatment, 825 IU/mL vs. no infection/treatment, 599 IU/mL, P = 0.09). There were significant differences in the levels of total IgE between infected and non-infected children within the treatment groups (infection/no treatment vs. no infection/no treatment, P < 0.001; infection/treatment vs. no infection/treatment, P < 0.001). Relatively few children (16%) had IgE levels below the upper range of normal for children living in industrialized countries [22] (age 7 years [<248 IU/mL], 18.9%; age 8 years [<280 IU/mL], 16.5%; age 9 years [<304 IU/mL], 17.3%; age 10 years [<328 IU/mL], 24.7%; age ≥11 years [<114 IU/mL], 5.3%). None of the *a priori *confounding factors or treatment group was associated with levels of total IgE at baseline in any of the 4 models (Table 2). Parameters of geohelminth infection were strongly positively associated with fold-increased levels (OR>1) of total IgE in all models: a) any geohelminth parasite (Model 1, P < 0.001; Model 4, P = 0.001); b) infections with *T. trichiura *(Model 2, P < 0.001); c) increasing infection intensities with *T. trichiura *(Model 3, test for trend across tertiles, P < 0.001); and d) increasing levels of anti-*A. lumbricoides *IgG antibodies (Model 4, test for trend across tertiles, P < 0.001).

**Table 2:** Relationship between baseline variables and levels of total IgE before treatment.

		Geometric mean total IgE at baseline							
Variable		Model 1		Model 2		Model 3		Model 4	
		Fold difference (95% CI)	p value	Fold difference (95% CI)	p value	Fold difference (95% CI)	p value	Fold difference (95% CI)	p value
Age (per year)		1.01 (0.94–1.10)	0.74	1.01 (0.94–1.09)	0.78	1.02 (0.94–1.10)	0.70	1.05 (0.97–1.12)	0.23
Sex (females relative to males)		0.83 (0.64–1.07)	0.15	0.84 (0.65–1.09)	0.20	0.85 (0.66–1.10)	0.22	0.86 (0.68–1.10)	0.24
Body Mass Index (per kg/m^2^)		1.01 (0.94–1.08)	0.81	1.01 (0.95–1.08)	0.73	1.01 (0.95–1.08)	0.68	1.00 (0.94–1.06)	0.98
Socioeconomic level (per unit score)		1.10 (0.95–1.27)	0.18	1.09 (0.94–1.26)	0.25	1.11 (0.96–1.28)	0.17	1.06 (0.93–1.21)	0.40
Crowding (per person/sleeping room)		0.98 (0.87–1.10)	0.74	0.98 (0.87–1.10)	0.74	0.97 (0.87–1.09)	0.66	1.00 (0.90–1.11)	0.99
Treated group (relative to untreated)		0.95 (0.73–1.24)	0.71	0.95 (0.73–1.23)	0.69	0.96 (0.74–1.25)	0.79	0.96 (0.75–1.23)	0.75
Geohelminth prevalence at t = 0 (present versus absent)									
Any		2.10 (1.60–2.75)	**<0.001**					1.55 (1.19–2.01)	**0.001**
*A. lumbricoides*				1.38 (0.99–1.91)	0.06				
*T. trichiura*				1.79 (1.31–2.46)	**<0.001**				
Hookworm				1.60 (0.99–2.60)	0.06	1.55 (0.95–2.52)	0.08		
*S. stercoralis*				0.77 (0.26–2.23)	0.62	0.71 (0.24–2.05)	0.52		
Geohelminth intensity (epg) at t = 0 (relative to no infection)									
*A. lumbricoides*^a^	1–8000					1.55 (1.05–2.30)	0.03		
	>8000					1.33 (0.85–2.08)	0.21		
*T. trichiura*^b^	1–500					1.32 (0.90–1.96)	0.16		
	>500					2.23 (1.44–3.43)	**<0.001**		
Anti-*A. lumbricoides *antibodies at t = 0									
(relative to lower tertile) IgG^c^									
	middle tertile							1.64 (1.21–2.23)	**0.001**
	upper tertile							3.51 (2.55–4.82)	**<0.001**

Statistical significance, after Bonferroni corrections, is inferred by: Model 1 (P < 0.05), Model 2 (P < 0.01), Model 3 (P < 0.008), and Model 4 (P < 0.02). Statistically significant P values are shown in bold for each model. P values for trend: ^a^0.08, ^b, c^<0.001.

### Factors associated with changes in total IgE

**Figure 2:**
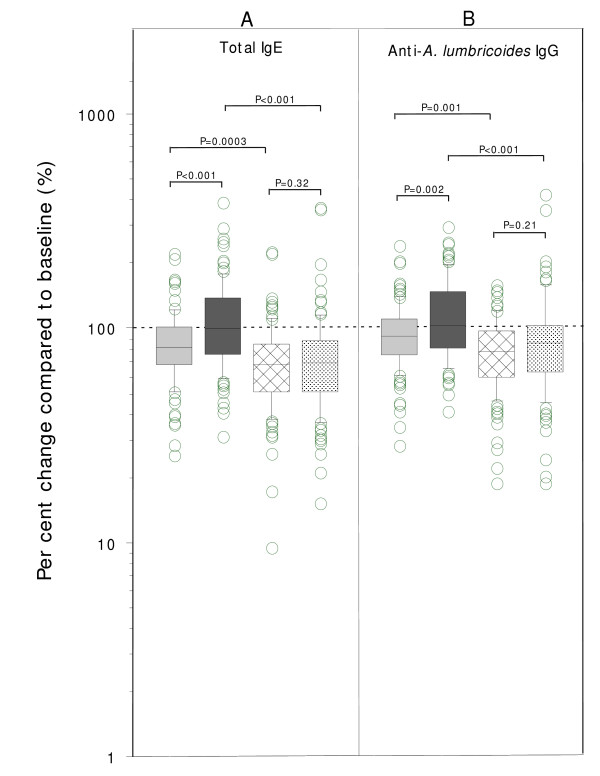
Effect of albendazole treatment on changes in levels of total IgE (A) and *Ascaris lumbricoides*-specific IgG (B) antibodies at 12 months compared to baseline. 100% indicates no change. Groups are: 1) infection/no treatment (light grey boxes), 2) no infection/no treatment (dark grey), 3) infection/treatment (hatched), and 4) no infection/treatment (checked). Box plots show median (middle line), interquartile range (box margins), 95% data range (bars), and outlying values (circles). Statistical significance in both Figures, after Bonferroni corrections (only the 4 comparisons shown were considered), is inferred by P < 0.0125.

Geometric mean levels of total IgE at 12 months and geometric mean percent changes in IgE levels (brackets) in the 4 study groups were: infection/no treatment, 1,045 IU/mL (79.5% or a posttreatment fall (-) of 20.5% compared to pretreatment levels); no infection/no treatment, 833 IU/mL (101.0% or +1.0%); infection/treatment, 999 IU/mL (63.8% or -36.2%); and no infection/treatment, 395 IU/mL (66.0% or -34.0%). Overall in the treatment and no treatment groups, IgE levels fell significantly by (-)35.1% (P < 0.0001) and (-)10.4% (P = 0.0004), respectively. Significantly greater declines in IgE levels were observed in the treatment groups compared to the no treatment groups for both non-infected (P < 0.001) and infected (P = 0.0003) children (Figure 2A). Anthelmintic treatment had relatively little impact on the proportion of children with levels of IgE within the normal range for children in industrialized countries [22]: (age 7 years [<248 IU/mL), 17.6%; age 8 years [<280 IU/mL], 21.5%; age 9 years [<304 IU/mL], 17.3%; age 10 years [<328 IU/mL], 33.8%; age ≥11 years [<114 IU/mL], 12.6%). The factors associated with changes in IgE levels are shown in Table 3. Receipt of albendazole treatment was strongly associated with falls (OR<1) in total IgE over the 12-month period in all models (Table 3). The following variables were also significantly associated with falls in IgE: a) infection with any geohelminth at baseline (Model 1, P = 0.01); b) increasing infection intensities with *A. lumbricoides *at baseline (Model 3, test for trend across tertiles, P = 0.05) and c) increasing levels of anti-*A. lumbricoides *IgG at baseline (Model 4, test for trend across tertiles, P = 0.02). Increases in IgE over the study period (OR>1) were associated with the presence of any geohelminth infection at 12 months (Models 1 and 4, P = 0.02), and increasing levels of anti-*A. lumbricoides *IgG at 12 months (Model 4, test for trend across tertiles, P = 0.03).

**Table 3:** Relationship between variables and geometric mean fold change in total IgE at 12 months compared to baseline.

Explanatory variable		Fold difference in geometric mean of fold change in total IgE from 0 to 12 months								
			Model 1		Model 2		Model 3		Model 4	
			Fold difference (95% CI)	p value	Fold difference (95% CI)	p value	Fold difference (95% CI)	p value	Fold difference (95% CI)	p value
Age (per year)			1.01 (0.94–1.10)	0.08	0.98 (0.95–1.00)	0.10	0.98 (0.95–1.00)	0.08	0.98 (0.95–1.00)	0.09
Sex (females relative to males)			0.97 (0.88–1.06)	0.48	0.96 (0.88–1.06)	0.42	0.96 (0.88–1.06)	0.44	0.97 (0.88–1.06)	0.50
Body Mass Index (per kg/m^2^)			1.00 (0.98–1.02)	0.89	1.00 (0.98–1.02)	0.95	1.00 (0.98–1.02)	0.98	1.00 (0.98–1.02)	0.89
Socioeconomic level (per unit score)			0.97 (0.92–1.02)	0.22	0.97 (0.92–1.02)	0.22	0.97 (0.92–1.02)	0.19	0.97 (0.93–1.02)	0.30
Crowding (per person/sleeping room)			0.98 (0.94–1.02)	0.28	0.98 (0.94–1.02)	0.31	0.98 (0.94–1.02)	0.31	0.98 (0.94–1.02)	0.26
Treated group (relative to untreated)			0.76 (0.69–0.84)	**<0.001**	0.75 (0.68–0.84)	**<0.001**	0.75 (0.68–0.84)	**<0.001**	0.77 (0.70–0.86)	**<0.001**
Geohelminth prevalence at t = 0 (present versus absent).	Any		0.88 (0.80–0.97)	**0.01**					0.89 (0.81–0.99)	0.03
	*A. lumbricoides*				0.95 (0.85–1.07)	0.38				
	*T. trichiura*				0.95 (0.84 1.07)	0.40				
	Hookworm				0.81 (0.67–0.96)	0.02	0.80 (0.67–0.96)	0.02		
	*S. stercoralis*				1.15 (0.78–1.69)	0.47	1.19 (0.81–1.75)	0.38		
Geohelminth prevalence at t = 12 (present versus absent).	Any		1.14 (1.02–1.26)	**0.02**					1.13 (1.02–1.26)	0.02
	*A. lumbricoides*				1.11 (0.98–1.26)	0.11				
	*T. trichiura*				1.04 (0.92–1.18)	0.49				
	Hookworm				1.04 (0.81–1.33)	0.76	1.08 (0.84–1.38)	0.56		
	*S. stercoralis*				0.92 (0.56–1.51)	0.74	0.92 (0.56–1.52)	0.76		
Geohelminth intensity (epg) at t = 0 (relative to no infection)	*A. lumbricoides*^a^	1–8000					0.94 (0.82–1.08)	0.40		
		>8000					0.86 (0.73–1.00)	0.05		
	*T. trichiura*^b^	1–500					1.06 (0.92–1.21)	0.45		
		>500					0.93 (0.79–1.10)	0.42		
Geohelminth intensity (epg) at t = 12 (relative to no infection)	*A. lumbricoides*^c^	1–8000					1.12 (0.94–1.35)	0.20		
		>8000					1.15 (0.93–1.42)	0.21		
	*T. trichiura*^d^	1–500					1.08 (0.93–1.25)	0.32		
		>500					1.03 (0.85–1.25)	0.76		
Anti-*A. Lumbri-coides *IgG t = 0^e^	middle tertile								0.87 (0.75–1.00)	0.05
	upper tertile								0.81 (0.68–0.96)	0.02
Anti-*A. lumbricoides *IgG t = 12^f^	middle tertile^g^								1.14 (0.99–1.31)	0.06
	upper tertile								1.22 (1.02–1.46)	0.03

Statistical significance, after Bonferroni corrections, is inferred by: Model 1 (P < 0.03), Model 2 (P < 0.006), Model 3 (P < 0.004), and Model 4 (P < 0.008). Statistically significant P values are shown in bold for each of the 4 models. P values for test for trend: ^a^0.05 ^b^0.61 ^c^0.13 ^d^0.55 ^e^0.018 ^f^0.025. ^g^Tertile boundaries are defined by the t = 0 data and applied also to the t = 12 data.

### Levels of anti-*A. lumbricoides *IgG antibodies

All children (100%) had positive serologic assays for anti-*A. lumbricoides *IgG before and after treatment. Per cent changes in levels of specific IgG in the 4 study groups (Figures 2B) were: infection/no treatment, 90.2% (-9.8%); no infection/no treatment 108.9% (+8.9%); infection/treatment, 74.4% (-25.6%); and no infection/treatment, 80.3% (-19.7%). Significantly greater reductions in levels of specific IgG antibodies were observed in treatment compared to no treatment groups (IgG, P≥0.001 for both infected and non-infected children).

Infections with *A. lumbricoides *and *T. trichiura*, but not hookworm or *S. stercoralis*, were positively associated with anti-*A. lumbricoides *IgG at baseline. The *A. lumbricoides *infection intensity stratum of 1–8,000 epg was associated with increased baseline antibody levels compared to the egg-negative group (1.5-fold increase, 95% CI 1.22–1.85, P < 0.001), and a similar but non-significant trend was observed for the *A. lumbricoides *infection stratum of >8,000 epg. Increased baseline antibodies were strongly associated with the heavy infection stratum (>500 epg) of *T. trichiura *infection, (1.55, 95% CI 1.23–1.95, P < 0.001). Tests for trend across strata of infection intensities for *A. lumbricoides *and *T. trichiura *were not statistically significant. Percent change in levels of anti-*A. lumbricoides *IgG antibodies was negatively associated with *A. lumbricoides *infection at baseline, but not at 12 months, and was not associated with the other geohelminth parasites. Antibody levels fell by a factor of 0.81 (95% CI 0.69–0.93, P = 0.004) in the >8,000 epg stratum of *A. lumbricoides *infection compared to egg-negative individuals.

## Discussion

The factors that determine levels of polyclonal IgE in populations living in the rural tropics are poorly understood but the presence of geohelminth infections and host genetic factors are likely to be important [10,11]. In this study, we have investigated the association between geohelminth infections and total IgE within the context of other known determinants of IgE (e.g. age, and nutritional and socioeconomic status) in a population of school children living in rural tropical communities in Ecuador where geohelminth infections are highly endemic [1,19] and have assessed the impact of repeated treatments with albendazole over the period of 12 months on IgE levels.

Levels of IgE at baseline were strongly positively associated with geohelminth infection and anti-*A. lumbricoides *IgG antibodies. Because of extensive immunologic cross-reactivity between antigens from different geohelminth parasites [21], levels of anti-*A. lumbricoides *IgG antibodies are likely to reflect past and current infections with geohelminth infections in general – levels of anti-*A. lumbricoides *antibodies at baseline were strongly associated with infections with *A. lumbricoides *and *T. trichiura*. Over the 12-month observation period during which two of the study groups received repeated anthelmintic treatments, fold falls in IgE levels were strongly independently associated with the receipt of anthelmintic treatment, any geohelminth infection at baseline, increasing infection intensities with *A. lumbricoides*, and increasing levels of anti-*A. lumbricoides *IgG antibodies. In contrast, fold increases in IgE levels over the observation period were associated with the presence of any geohelminth infection at 12 months and increasing levels of anti-*A. lumbricoides *IgG at 12 months. These findings suggest that geohelminth infections are important determinants of polyclonal IgE levels and that anthelmintic treatments are capable of significantly reducing levels of IgE over a period of 12 months but that, independent of treatment, active infections and chronic and continued exposures (indicated by post-treatment levels of anti-*A. lumbricoides *IgG) are important determinants of elevated IgE levels post-treatment.

To our knowledge, previous studies have not examined specifically the association between total IgE and intestinal helminth infections in endemic populations and the effects of anthelmintic treatment on IgE levels in the context of other important risk factors. The study sample was derived from an intervention study that examined the effects of repeated anthelmintic treatments on atopy and clinical allergy over a period of 12 months [19]. Random samples of children were selected from children completing follow-up into four groups defined by geohelminth infection status and treatment allocation. The random samples of children were not selected within schools, the original unit of randomization, because of the small sizes of schools. If there were appreciable differences between schools then this would increase the likelihood of imbalance between the samples. However, this does not seem to have occurred, because the four groups were comparable with respect to the *a priori *confounders (i.e. age, sex, and crowding) and these characteristics were similar to that observed in the original pre-treatment study population [19] that recruited a high proportion of all children attending 68 rural schools. Socioeconomic level was lower in the infected groups compared to non-infected groups – this may not be surprising given the known associations between these infections and poverty [5,13,16]. Another important strength of the study is the collection of data on potential confounding factors and controlling for these in the analysis. Residual confounding by socioeconomic level cannot be excluded because this variable was controlled for in the analysis using a score that is unlikely to capture the complexity of social and economic disparities within a population. However, residual confounding would seem unlikely because there was no indication of an association between socioeconomic score and the IgE parameters in the multivariable models. Our findings are likely to be relevant to schoolchildren living in areas of the rural tropics where geohelminth infections are highly endemic.

Investigations of IgE in helminth-exposed populations or infected patient groups have examined the effects on IgE levels of anthelmintic treatment [16-18] and the effects of migration on IgE levels in individuals that migrate from endemic to non-endemic countries. Studies of the effects of anthelmintic treatment on IgE levels have shown that levels of IgE remain highly elevated 2 to 3 years after providing effective anthelmintic treatment [16,17]. A study of 34 expatriates with filarial helminth infections and provided effective anthelmintic treatment showed that the mean IgE level fell to within normal limits after a median of 33 months [18]. Studies of migrants from helminth-endemic countries to non-endemic countries have shown that IgE levels take at least 10 years to decline to those observed in the native populations [23,24]. In the current study, we observed declines in total IgE of 34.0% and 36.2% in the non-infected and infected treatment groups, respectively, and geometric mean levels of IgE remained highly elevated at 12 months after treatment. A fall was observed in the infected non-treatment group (of 20.5%) that may be explained by treatment contamination. IgE levels did not change in the non-infected non-treatment. There are likely to be differences between different study populations that depend on the effectiveness of anthelmintic treatment for the parasites studied and whether individuals remain exposed to helminth infections (i.e. by continued residence in an endemic area). Together these studies show that IgE levels may remain elevated for long periods of time after treatment even though the life of IgE in the circulation is approximately 3 to 4 days, and indicate that helminth infections have long-lasting effects on host immunity.

Possible explanations for the longevity of the IgE response include. 1) The continued exposure to infectious parasite stages in the environment or non-curative chemotherapy. In the current study all children had significant levels of anti-*A. lumbricoides *IgG both pre- and post-treatment that may indicate intense and continued transmission of geohelminth infections over the course of the study. ; 2) The immune system may be programmed for Th2 responses in early life through exposure to maternal helminth infections, early childhood infections [25], or other Th2-skewing environmental exposures and the effect may be long-lasting. For example, newborns of Ethiopian immigrant mothers with high level of IgE are born with relatively high levels of IgE in cord blood that are proportionate to maternal values [26]. 3) Certain populations may maintain high levels of IgE because of genetic predisposition.

Levels of IgE reflect a complex interaction between genetic factors and environmental exposures. In the United States, ethnicity appears to be the most important determinant of IgE levels although this is likely to reflect a number of genetic and environmental effects [9]. A study among a genetically isolated population in Nepal where *A. lumbricoides *and hookworm infections were highly endemic provided evidence that more than 50% in variability in total IgE levels was attributable to genetic factors [11]. Studies of highly parasitized Aboriginal Australians have provided evidence that a polymorphism in the high-affinity IgE receptor and HLA-DR genes explained 12.4% [10] and 33% [27], respectively, of the variation in serum total IgE. Unfortunately, we were not able to investigate the role of genetic factors in this study. However, the fact that total IgE levels are elevated in almost all individuals infected with geohelminth parasites [12] indicates that geohelminth infections are potent environmental determinants of IgE.

## Conclusion

Our data provide strong evidence for geohelminth infections being an important determinant of the highly elevated levels of IgE observed in a population of school children living in an area of the rural tropics endemic for these parasites. Parameters of geohelminth infection were strongly associated with pretreatment IgE levels and also with the magnitude of the decline in IgE after anthelmintic treatment. Repeated anthelmintic treatments resulted in significant reductions in levels of total IgE of 34.0 to 36.2%. However, the failure to reduce levels of total IgE to 'normal' values in a high proportion of children may be explained by a combination of factors that may include continued exposure to parasites in a contaminated environment, programming for a Th2-skew in early life and long-lasting immunologic effects induced by helminth parasites, and other unidentified environmental and genetic factors.

## Abbreviations

ELISA: Enzyme-Linked ImmunoSorbent Assay; HLA: human leukocyte antigen; IgE: immunoglogulin E; IgG: immunoglobulin G; IL-interleukin; IU/mL: international units per mililitre; OR: odds ratio; epg: eggs per gramme of faeces; Th2: T helper 2.

## Competing interests

The authors declare that they have no competing interests.

## Authors' contributions

PJC designed the study and supervised the conduct of the study, drafted the manuscript and prepared it for publication. NA performed the statistical analyses. ALM and SB performed the laboratory analyses. MC and MV supervised the field study and collection of clinical samples. GEG was involved in study design and drafting the manuscript. All authors read and approved the final manuscript.
